# Nocturnal Cleaning Interactions Between the Giant Moray (*Gymnothorax javanicus*) and the Clear Cleaner Shrimp (*Urocaridella antonbruunii*)

**DOI:** 10.1002/ece3.70589

**Published:** 2024-12-23

**Authors:** Daniel M. Cryan, Kelsey M. Vaughn, Eleanor M. Caves

**Affiliations:** ^1^ Odum School of Ecology University of Georgia Athens Georgia USA; ^2^ Department of Ecology, Evolution, and Organismal Biology Brown University Providence Rhode Island USA

**Keywords:** cleaning mutualism, niche partitioning, nocturnal, signaling

## Abstract

We observed a novel, nocturnal cleaning interaction between a cleaner shrimp (Genus *Urocaridella*) and the giant moray eel ( *Gymnothorax javanicus* ) on a lagoonal patch reef in Moorea, French Polynesia. Over the course of an 85‐min foraging bout (recorded on video by a snorkeler), we observed three separate, stereotyped cleaning interactions between *G. javanicus* and a cleaner shrimp in the genus Urocaridella (which surveys of Moorea biodiversity previously visually identified as *Urocaridella antonbruunii* ). During these interactions, the shrimp would slowly crawl along one of the eel's flanks towards its head, enter its mouth, emerge on the other side of its head, then crawl back towards the reef along the eel's opposite flank, often causing it to jolt in response. On each of the visits, the moray spent roughly 9–12 min at the cleaning station and was observed being cleaned for a total of 62 s. Although this was a chance observation of only a few instances of cleaning, it may have several important implications for our understanding of the behavioral ecology of cleaning mutualisms, including (1) indicating potential temporal trade‐offs between being cleaned and foraging in eels, (2) suggesting a degree of temporal niche partitioning among sympatric cleaner species and (3) updating our understanding of cleaner‐client communication, given the nocturnal nature of our observations.

## Graphical abstract

We observed a novel nocturnal cleaning interaction between a giant moray eel and a clear cleaner shrimp. This observation is unique since cleaning interactions are primarily visually mediated and thus typically confined to daylight hours. The existence of nocturnal cleaning therefore implies potential temporal niche partitioning of cleaning mutualisms, as well as alternative signaling modalities for mediating cleaning interactions, such as tactile signaling.

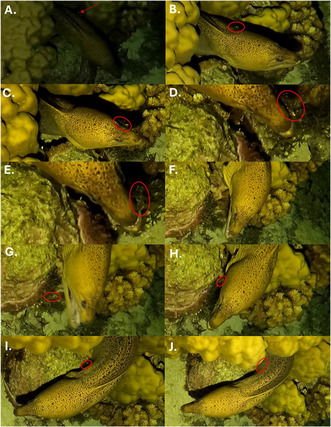

## 1 Introduction

Cleaning mutualisms involve a ‘cleaner’ (usually a shrimp or small fish) removing ectoparasites or dead skin from a ‘client’ (typically a larger fish). Clients benefit from reduced parasite loads (Grutter 1999; Grutter and Lester 2002) and tactile stimulation (Soares et al. 2011), which may yield secondary fitness benefits like enhanced growth (Clague et al. 2011) and improved cognitive performance (Binning et al. 2018). Cleaners benefit in these interactions from access to food resources and protection from predators, which often refrain from preying on cleaners due to the benefits gained from cleaning (Gingins, Roche, and Bshary 2017). Cleaning interactions occur worldwide in marine environments but are most common in the tropics, among shrimps in the families *Palaemonidae* and *Lysmatidae*, wrasses in the family *Labridae*, and gobies in the genus *Elacatinus*. In the diverse communities where cleaning typically occurs, multiple cleaner species often coexist and may compete for clients.

To reduce competition for clients, some cleaners have evolved distinct cleaning niches. For example, cleaners may divide their niches based on client species, only cleaning distinct portions of the client fish community and thereby reducing competition (Adam and Horii 2012). Yet studies of cleaning mutualism network structure often show high levels of nestedness at the community level, meaning sympatric cleaners often interact with the same core group of clients (Sazima et al. 2010). This suggests other potential axes of niche differentiation for cleaners, such as habitat, behavior, and time of day. For example, one study found that three sympatric species of cleaner wrasse did not vary in client composition, but occupied distinct microhabitats, defined largely by depth and the presence of live coral (Côté and Brandl 2021). Another study found that two closely related species of cleaner wrasse used different movement strategies, with one exhibiting high site fidelity and the other roaming widely (Oates, Manica, and Bshary 2010). Overall, the complexity of cleaning interactions offers multiple opportunities for niche differentiation, yet few studies have fully examined these dynamics. Specifically, temporal partitioning of cleaning niches (i.e., cleaning at different times of the day) has received relatively little attention.

Visual signaling often plays a key role in mediating complex cleaning behaviors. Both cleaners and clients may signal to each other to indicate their intent to participate in cleaning interactions. For client fish, this visual signaling typically involves color changes (Caves, Green, and Johnsen 2018) or posing (i.e., the fish altering the presentation of its body to facilitate cleaning, such as opening its mouth, flaring its gills, spreading its fins, or adopting a vertical posture) (Côté, Arnal, and Reynolds 1998). Cleaner signals can vary by species. For example, the cleaner shrimp *Ancylomenes pedersoni* signals to its clients by whipping its antennae (Caves, Green, and Johnsen 2018), while the cleaner wrasse *Labroides dimidiatus* signals to its clients by exhibiting undulating ‘dancing’ behavior (Potts 1973). These signals help reduce potential conflicts between cleaners and clients by allowing parties to recognize one another and engage in cooperative behavior. Interestingly, since most observations of cleaning interactions have been during the day, demonstrations of signaling in cleaning interactions are largely visual (Becker and Grutter 2005; Chapuis and Bshary 2010; Caves, Frank, and Johnsen 2016). Nocturnal cleaning mutualisms have been reported (Bos and Fransen 2018) but are poorly studied compared to diurnal cleaning mutualisms. This pattern may partially be the result of a larger diurnal bias in field behavioral studies, due to the greater difficulties of conducting marine fieldwork at night.

## 2 Methods

### 2.1 Nocturnal Foraging Observations of *Gymnothorax javanicus*

On June 11, 2023, while conducting daytime fish surveys in the Maharepa Lagoon, on the north shore of Moorea, French Polynesia, we located an adult giant moray eel ( *Gymnothorax javanicus* ), approximately 2 m in total length, resting in a coral bommie and marked its location on GPS and with a small buoy. We returned later in the evening, around 6 PM (i.e., shortly after sunset) to observe the moray's nocturnal foraging behavior. For over 85 min, an individual snorkeler followed the moray as it hunted in the lagoon, recording its behaviors using a Go Pro Hero 9 underwater camera and a yellow light (Suptig, Shenzhen Runshengxing Technology Co. Ltd. Shenzen, China). Later, we reviewed the footage and constructed an ethogram for the focal moray. We classified moray behavior into six stereotyped categories: swimming (actively moving around in the open), searching (moving underneath a coral bommie, presumably looking for prey), striking (trying to bite another fish), inducing fleeing (causing a potential prey species to swim away), resting (not actively moving), and repositioning (changing orientation while resting, without leaving its resting spot). Surprisingly, we also observed the eel being cleaned by a shrimp, and so added three additional behavioral categories to our ethogram: gaping its mouth (opening its mouth wide, perhaps to facilitate cleaning), getting cleaned (physical contact between eel and shrimp, presumably during which the cleaner is removing dead skin and parasites from the eel), and jolting its body (twitching/shuddering its body, perhaps in response to cheating by the shrimp). Note that the mouth gaping we recorded appeared more pronounced than the typical shallow mouth gaping commonly observed in resting morays. We also recorded any instances in which the moray was not visible.

## 3 Results

At the beginning of the observation, the moray exhibited active foraging behavior, alternating between swimming in the open and searching for prey underneath coral bommies. Overall, active swimming constituted roughly 18% of the moray's total activity, while 9% of the moray's time was spent searching for prey. Additionally, the moray was not visible for approximately 7% of the total observation (Table 1). While foraging, we observed 12 instances in which the moray induced flight in other reef fish (i.e., a potential anti‐predator response). These include 10 fish species, representing 7 families and a range of trophic groups (Appendix: Table A1). We also observed two predation attempts (strikes) by the moray towards two species of reef fish: *Epinephalus tauvina* (greasy grouper) and *Chaetodon lunulatus* (redfin butterflyfish), neither of which was successful. However, the actual number of strikes may be higher, as the moray was partially obscured from view while searching for prey within the reef.

**TABLE 1:** Summary of giant moray nocturnal foraging behaviors.

Behavior	Description	*N*	Mean Duration	SE Duration	Total Duration	Proportion of Total Activity
Swim	Moray actively moves around in the open	23	41.2	7.62	947	0.184
Search	Moray moves underneath a coral bommie	15	31.1	5.35	467	0.091
Induce fleeing	Moray causes a potential prey species to swim away	12	2.3	0.33	27	0.005
Strike	Moray tries to bite another fish	2	1.0	0.00	2	0.000
Rest	Moray not actively moving	15	250.9	105.3	3763	0.731
Reposition	Moray changes orientation while resting, without leaving the reef	6	17.5	3.75	105	0.020
Mouth agape	Moray opens its mouth wide	5	15.8	4.86	79	0.015
Get cleaned	Moray has its dead skin/parasites removed by the cleaner shrimp	3	20.7	3.33	62	0.012
Jolt	Moray twitches or shudders its body	9	2.0	0.33	18	0.003
Not visible	Moray temporarily not visible	25	15.2	2.12	379	0.074

*Note:* An overview of the behaviors we observed during the moray's nocturnal foraging bout. The mean, standard error, and total duration of each behavior are given in seconds. Note that many behaviors occur simultaneously with other behaviors (e.g., repositioning, gaping its mouth, getting cleaned, and body jolting all occur while the moray is at rest); thus, not all behaviors are mutually exclusive.

**FIGURE 1:**
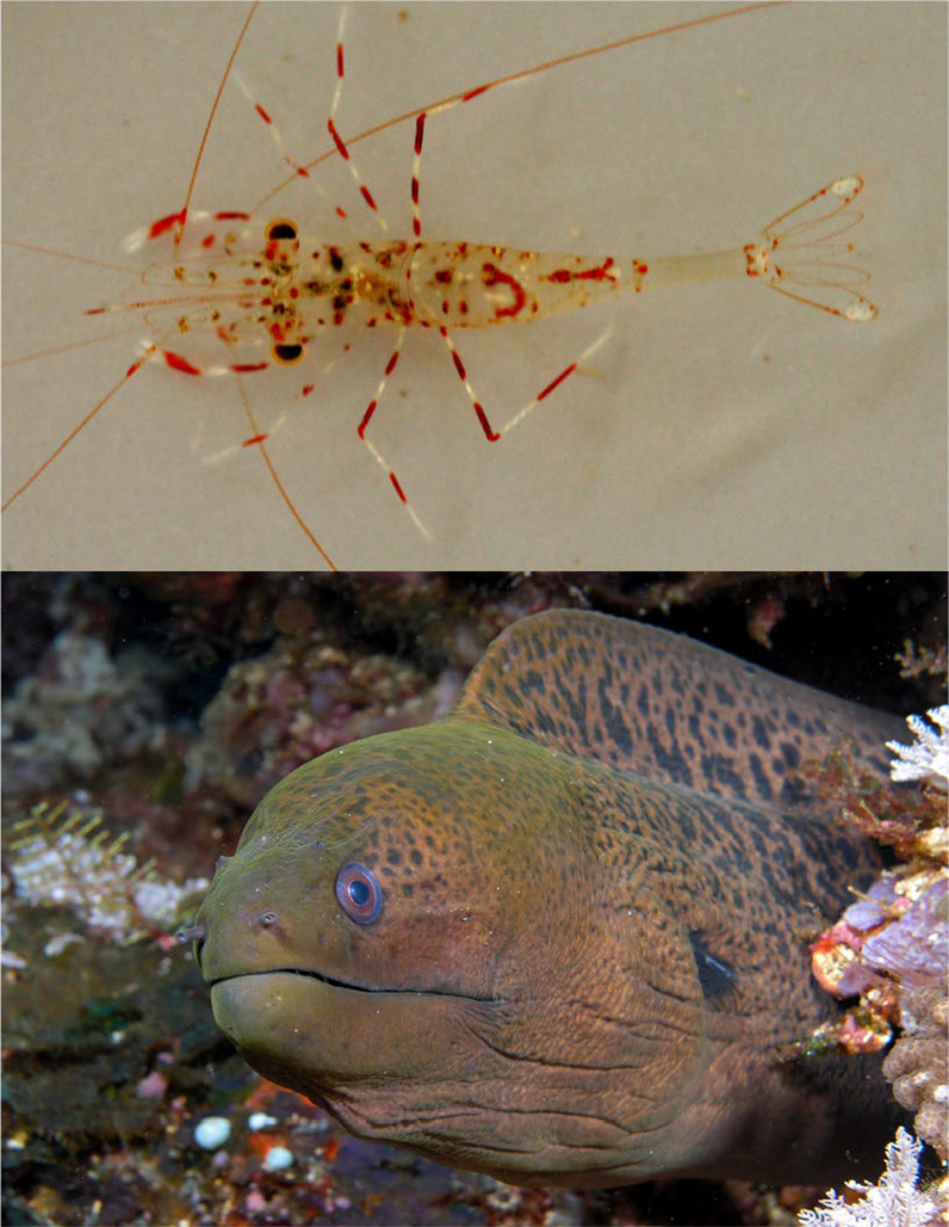
*Urocaridella antonbruunii* and *Gymnothorax javanicus* . Top: A clear cleaner shrimp, *U. antonbruunii* , photographed by Poupin J. & Corbari L in Nuku Hiva, French Polynesia; Wikimedia Commons. Bottom: A giant moray eel, *G. javanicus* , photographed by Nick Hobgood in Komodo, Indonesia; Wikimedia Commons.

Most of the moray's time (73% of its total activity) was spent resting at coral bommies. Two thirds of these were brief rests (i.e., 1 min or less), while the remaining third were extended rests (range: 2–24 min). The most notable behavior we observed during the moray's rest periods was a novel, nocturnal cleaning interaction with the clear cleaner shrimp ( *Urocaridella antonbruunii* ) (Figure 1).

### 3.1 Nocturnal Cleaning by *Urocaridella antonbruunii*

At around 70 min into our focal eel follow, we first observed the moray getting cleaned by the shrimp. Overall, we recorded a total of 61 s of cleaning behavior, separated over three interactions (range: 14–24 s) that were on average 20.7 s long (Table 1). Notably, the moray visited the shrimp cleaning station three times during our observation. Although no cleaning was observed during the first visit, the moray spent over 12 min resting there, repositioning itself once. It then returned to the station approximately 37 min later. During this second visit, the moray rested for nearly 9 min, repositioned itself once, and held its mouth agape for 10 s. Towards the end of this rest, the shrimp cleaned the moray, after which it exhibited a body jolt and left the station 30 s later, swimming to a nearby patch reef. Less than 3 min later, the moray returned to the station for another nine‐minute stay. During this final visit, the moray repositioned itself once, was cleaned by the shrimp less than a minute later, then held its mouth agape on three occasions, before being cleaned again. The moray exhibited body jolts after these cleaning events and left the station shortly thereafter. Overall, the moray spent over 30 min at the shrimp cleaning station, comprising nearly 36% of its total observed activity time.

**FIGURE 2:**
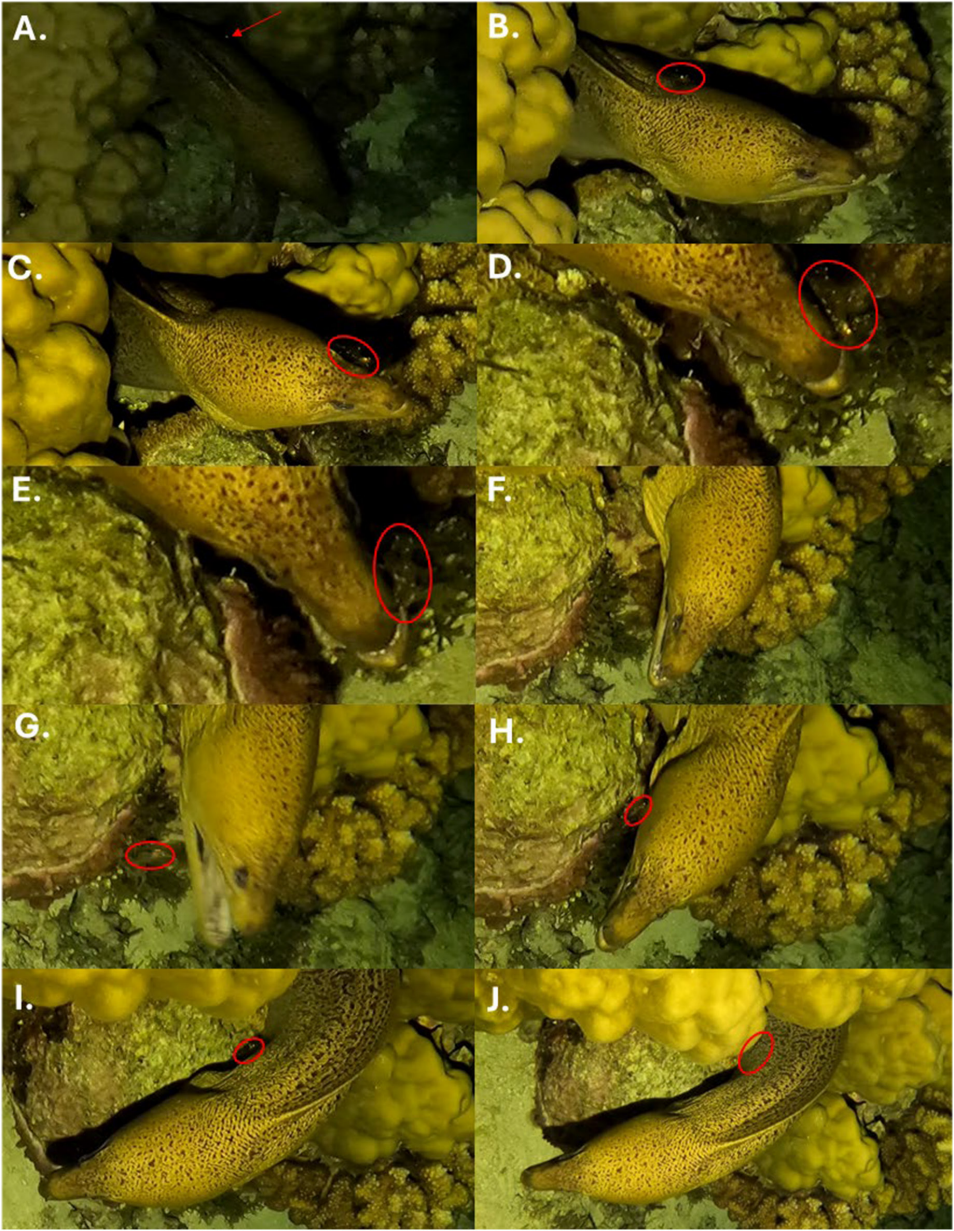
Stereotyped cleaning interaction between *U. antonbruunii* and *G. javanicus* . A series of screen captures showing key moments in a typical nocturnal moray‐shrimp cleaning interaction. The shrimp was first spotted via its eye shine [(A), eye shine indicated with red arrow]. The shrimp then made its way along one of the moray's flanks (B), onto its head (C), towards the tip of its nose (D), where it then entered the moray's mouth (E). The shrimp remained in the moray's mouth for a couple seconds (F), before popping out the other side, often causing the eel to jolt in the process (G). Finally, the shrimp landed on the eel's opposite flank (H), crawled back along its side (I), before disappearing back into the reef (J). The cleaner shrimp is indicated with a red circle.

Cleaning interactions followed a distinct pattern. During each interaction, the shrimp gradually made its way forward, along one of the moray's flanks, moving towards its head. Next, the shrimp entered the eel's mouth or went onto the tip of its nose, then moved to the other side of the eel's head, before slowly crawling back along its opposite flank, returning to the hole in the reef from which it emerged. These interactions often seemed to induce body jolts in the moray, either immediately, or 2–6 s after cleaning interactions (Figure 2). The moray was also observed with its mouth agape several times while at the shrimp cleaning station, sometimes shortly before or after a cleaning interaction. Finally, the moray repositioned itself multiple times while at the shrimp cleaning station, often before or between cleans. While this repositioning could be random (it occurred in the absence of visible cleaning), it may have also served to facilitate access for the cleaner to different parts of the moray's body.

While at the cleaning station, we recorded four instances of the moray gaping its mouth, three instances of the moray repositioning itself (once during each visit), and four body jolts (often shortly after being cleaned). Interestingly, we also observed one instance of mouth gaping, three instances of repositioning, and five body jolts while the moray was resting for around 24 min at a different coral bommie. Although we did not observe cleaning interactions at this section of the reef, the presence of ancillary behaviors commonly associated with cleaning suggests that additional cleaning interactions (at additional cleaning stations) may have occurred. This is plausible, since the low light conditions and our distance from the moray for most of the observation made it difficult to spot the shrimp, which are only 3–4 cm long, and mostly transparent. Additionally, the moray's body was often partially obscured, and these shrimps typically reside within the reef's recesses, suggesting that cleaning activity may have occurred entirely out of view. Indeed, in the instances where we did observe cleaning, we were only able to detect the shrimp by the shine of its eyes as it emerged onto the moray's head (e.g., Figure 2B).

## 4 Conclusion

Our observation has several potentially interesting implications for our understanding of the ecology of marine cleaning mutualisms. Although limited in scope, (i.e., we only followed one moray), the rarity of other nocturnal observations of cleaning behavior makes this a valuable data point. Additionally, there are relatively few studies on the trophic ecology of moray eels compared to other coral reef predators, which is surprising given that morays are ubiquitous on reefs worldwide and one of the few fish families subject to relatively minor fishing pressure (and thus may play an increasingly important role in coral reef food webs under scenarios of heightened overfishing). Due to their cryptic and largely nocturnal nature, direct observations of moray foraging behavior are rare. Our observation supports common notions about the moray's trophic role, as an active, nocturnal hunter that targets fish hiding within the reef. It is notable that the moray spent over a third of its overall time at the shrimp cleaning station, with many of its longer rest periods occurring there. Thus, there may be a tradeoff between being cleaned and foraging for these eels, as has been reported for some diurnal reef fish (Adam 2012).

The most significant implications of our observations involve the nocturnal cleaning interactions with *U. antonbruunii* . First, the nocturnal nature of the interactions may suggest a degree of temporal niche partitioning of cleaning mutualisms on these reefs. Collectively, our field team has spent hundreds of daylight hours on this reef, and we have never observed this cleaner shrimp operating in the daylight, suggesting their apparent absence in daylight hours. On Moorea's reefs, cleaner wrasse (Genus Labroides) are the dominant diurnal cleaners, and we've often observed them cleaning giant morays during the day. However, these wrasses are not active at night, which leaves open a cleaning niche for the nocturnal shrimp to fill. This has implications for our understanding of the mechanisms that enable the existence of highly diverse communities on coral reefs. Specifically, several mechanisms have been proposed as underlying the coexistence of multiple cleaner species on one reef, including spatial segregation, selectivity for certain client species, or motivation of clients to visit certain cleaners (Quimbayo et al. 2018; Côté and Brandl 2021). Temporal separation is another potential mechanism, but there is little available evidence for this; in fact, currently the only study to investigate temporal niche partitioning in cleaning mutualisms found that sympatric cleaner shrimp and cleaner fish in the Caribbean clean at the same times of day (Titus and Daly 2015). Our observation suggests that indeed, temporal separation may operate between sympatric cleaner species on some reefs.

Second, this observation challenges prevailing hypotheses regarding the mechanisms underlying how cleaners and clients identify one another as beneficial partners and choose to interact. Current evidence suggests that marine cleaning mutualisms involving both fish and shrimp as cleaners are primarily mediated by visual signals. These signals play a crucial role in mediating interactions between cleaners and clients, particularly predatory clients with whom interacting poses a potential risk (reviewed in Caves 2021). However, the nocturnal nature of the interactions we observed likely precludes visual signaling, or at least means other modalities may be more effective for signaling. Interestingly, we observed the moray with its mouth agape several times while at the cleaning station. Client fish often signal cleaners by opening their mouths widely and flaring their operculum (Stummer et al. 2004). However, since these interactions occurred at night, the moray's gaping mouth may have served less as a signal per se, and more to facilitate access to cleaning. Still, there were several instances of the moray widely gaping its mouth when it was not visibly being cleaned. So, it is possible the eel intended to signal the cleaner, even if its signal was ineffective, or that the mouth gaping behavior simply served a different function in this context. Alternatively, it could be that cleaners and clients recognize one another through some other sense, such as touch. Indeed, some species of cleaner wrasse use tactile stimulation (in addition to visual signals) to mediate interactions with their clients (Bshary and Würth 2001; Grutter 2004; Soares et al. 2011). Tactile signaling has also been proposed to play a role in mediating some shrimp‐involved cleaning interactions, particularly in low‐light environments where visual signaling is not possible (Moura et al. 2019).

Finally, a lack of field observations conducted at night has likely limited our understanding of important nocturnal ecological dynamics. Nocturnal cleaning interactions have been recorded before in shrimps of the Genus Urocaridella, but only on sleeping rabbitfish (Family Siganidae) (Bos and Fransen 2018). What distinguishes our observation from the former is the active nature of the interaction and intentionality of both partners. The eel visited the cleaning station multiple times over the course of its foraging bout, spending considerable time there on each visit. Likewise, the shrimp approached and even entered the eel's mouth, behaviors observed previously in what are considered ‘obligate’ cleaning interactions. Obligate cleaning interactions are those in which the cleaner receives most of its food from cleaning; in which aspects of the cleaner's morphology and ecology are believed to be adapted to cleaning; and which have the greatest impact on the ecology and health of clients (Vaughan et al. 2017). If indeed we are inadvertently unaware of a whole class of obligate cleaning interactions—those occurring at night—we have a very incomplete picture of this ecologically important class of mutualistic interaction. This issue may be relevant to multiple taxa, as cleaning is found in birds, mammals, fish, and crustaceans, but across all these systems, nocturnal cleaning is highly underexplored. Overall, our observation highlights the value of nocturnal behavioral observations, which despite their logistical difficulties can often yield novel ecological discoveries and insights.

## Author Contributions

**Daniel M. Cryan:** conceptualization (equal), data curation (lead), formal analysis (lead), investigation (lead), methodology (equal), project administration (lead), supervision (lead), visualization (lead), writing – original draft (lead), writing – review and editing (supporting). **Kelsey M. Vaughn:** conceptualization (supporting), funding acquisition (lead), investigation (equal), methodology (equal), project administration (supporting), resources (equal), visualization (supporting), writing – review and editing (equal). **Eleanor M. Caves:** conceptualization (equal), investigation (supporting), project administration (supporting), supervision (supporting), visualization (supporting), writing – original draft (supporting), writing – review and editing (lead).

## Conflicts of Interest

The authors declare no conflicts of interest.

## Data Availability

Analyses reported in this article can be reproduced using the data provided by Cryan et al. 2024.

## Appendix

**TABLE A1:** Summary of fish species that fled the giant moray. An overview of the fish species that swam away from the moray while it was foraging (i.e., a potential ant‐predator response). The mean duration of each fleeing behavior is given in seconds. Family and trophic information are from FishBase.

Scientific name	Common name	Family	Trophic group	*N*	Mean duration
*Cephalopholis argus*	peacock grouper	Serranidae	Piscivore	2	2.5
*Epinephalus merra*	honeycomb grouper	Serranidae	Piscivore	1	1
*Epinephalus tauvina*	greasy grouper	Serranidae	Piscivore	1	1
*Sargocentron microstoma*	smallmouth squirrelfish	Holocentridae	Piscivore	1	2
*Sargocentron tiere*	blue lined squirrelfish	Holocentridae	Piscivore	1	4
*Pristiapogon exostigma*	oneline cardinalfish	Apogonidae	Piscivore	2	3.5
*Abudefduf sexfasciatus*	scissortail sergeant	Pomacentridae	Planktivore	1	2
*Chaetodon lunulatus*	redfin butterflyfish	Chaetodontidae	Corallivore	1	1
*Cheilinus chlorourus*	floral wrasse	Labridae	Invertivore	1	1
*Ctenochaetus striatus*	striated surgeonfish	Acanthuridae	Herbivore	1	3
